# Polymorphism rs2327430 in TCF21 predicts the risk and prognosis of gastric cancer by affecting the binding between TFAP2A and TCF21

**DOI:** 10.1186/s12935-024-03343-z

**Published:** 2024-05-07

**Authors:** Xinyi Zhou, Kuan Shen, Shuqing Cao, Pengyu Li, Jian Xiao, Jiacheng Dong, Quan Cheng, Li Hu, Zekuan Xu, Li Yang

**Affiliations:** Department of General Surgery, The First Affiliated Hospital of Nanjing Medical University, 300 Guangzhou Road, Nanjing, Jiangsu 210029 China

**Keywords:** Gastric cancer, Single nucleotide polymorphism, Transcription factor 21, Transcription factor AP-2 alpha

## Abstract

**Background:**

Single nuclear polymorphisms (SNPs) have been published to be correlated with multiple diseases. Transcription Factor 21 (TCF21) is a critical transcription factor involved in various types of cancers. However, the association of TCF21 genetic polymorphisms with gastric cancer (GC) susceptibility and prognosis remains unclear.

**Methods:**

A case-control study comprising 890 patients diagnosed with GC and an equal number of cancer-free controls was conducted. After rigorous statistical analysis, molecular experiments were carried out to elucidate the functional significance of the SNPs in the context of GC.

**Results:**

TCF21 rs2327430 (OR = 0.78, *P* = 0.026) provides protection against GC, while rs4896011 (OR = 1.39, *P* = 0.005) exhibit significant associations with GC risk. Furthermore, patients with the (TC + CC) genotype of rs2327430 demonstrate a relatively favorable prognosis (OR = 0.47, *P* = 0.012). Mechanistically, chromatin immunoprecipitation assay and luciferase reporter assay revealed that the C allele of rs2327430 disrupts the binding of Transcription Factor AP-2 Alpha (TFAP2A) to the promoter region of TCF21, resulting in increased expression of TCF21 and inhibition of malignant behaviors in GC cells.

**Conclusion:**

Our findings highlight the significant role of TCF21 SNPs in both the risk and prognosis of GC and provide valuable insights into the underlying molecular mechanisms. Specifically, the disruptive effect of rs2327430 on TCF21 expression and its ability to modulate malignant cell behaviors suggest that rs2327430 may serve as a potential predictive marker for GC risk and prognosis.

**Supplementary Information:**

The online version contains supplementary material available at 10.1186/s12935-024-03343-z.

## Introduction

Despite the availability of numerous potential therapies, GC remains the fifth most common cancer in terms of incidence and the fourth leading cause of cancer-related death worldwide, posing a significant threat to human health [1]. According to recent data, GC was responsible for 397,000 new cases and approximately 290,000 deaths in China in 2016 [2], most of whom were diagnosed at advanced stages. Subsequent studies have consistently demonstrated that early treatment could have a positive impact on the prognosis of GC patients [3]. Therefore, early screening for GC among populations with high risk could contribute to improving the patient’s prognosis and overall survival [4, 5].

Single nucleotide polymorphism (SNP) is a common form of heritable DNA variation linked to tumor development and progression [6, 7]. Numerous studies have suggested that SNPs could assist in detecting malignant tumors at an early stage, predicting the risk in key populations, and assessing the prognosis of patients [8]. For instance, the rs3807213 variant genotypes of IFRD1 have been associated with increased susceptibility to GC [9]. Moreover, the rs1141023 variant located in TRIM59 has been linked to a rising predisposition to GC, potentially correlated with early-stage GC [10]. The effects of transcription factors on the malignant cell behaviors of GC have also been widely reported [11, 12]. However, comprehensive empirical support regarding the effects of SNPs located in transcription factors in GC is still lacking. Thus, our study aimed to explore the role of TCF21 SNPs in GC.

The TCF21 gene encodes a basic helix-loop-helix transcription factor at 6q23.2 [13, 14]. TCF21 is downregulated and plays protective roles in various malignant tumors, such as hepatocellular carcinoma, ovarian cancer, and renal tumors [13, 14]. Yang et al. demonstrated that TCF21 was downregulated and suppressed malignant behaviors through the AKT pathway in GC [15]. Furthermore, researchers have suggested that rs12190287 located in TCF21 is associated with a poor prognosis in breast cancer [16]. To investigate the function of SNPs located in TCF21, we selected four tagSNPs and examined their relationship with GC.

## Materials and methods

### Study subjects

All participants included in the study comprised 890 GC cases histopathologically confirmed by at least two pathologists and 890 cancer-free controls without any history of malignancy. All patients received medical treatment at the First Affiliated Hospital of Nanjing Medical University between February 2012 and March 2020. Exclusion criteria and detailed definitions are provided in a previous study [1].

### SNP selection

Several studies suggest that selecting tagSNPs instead of all SNPs in a given interval does not result in a substantial loss of power, and it is reasonable to choose potentially functional SNPs and a selection of tagSNPs as objects of a study [17, 18]. All tagSNPs of the TCF21 gene full-length and upstream 2000 bp in chromosome 6, position 133,887,113–133,895,553 in the Chinese population were selected on the grounds of the 1000 Genomes Project and HaploView 4.2 software under the following conditions: r2 > 0.8 and a minor allele frequency (MAF) > 0.05. Previous studies have suggested that SNPs in nonsynonymous mutated regions, splice site regions, and untranslated regions (UTRs) have a higher potential to have functional effects than SNPs in intronic regions, as introns are typically removed during the formation of mature mRNA [19, 20]. Therefore, we excluded SNPs in the intron region and identified five SNPs [rs12190287 (3’-UTR), rs2327429 (promoter), rs2327430 (promoter), rs3734281 (3’-UTR), rs4896011 (3’-UTR)] that potentially influence the expression of TCF21. Based on respective genomic locations, the JASPAR database and miRNASNP website were used to analyze the function of sites situated in promoter regions or 3’UTRs, respectively (Fig. S1A-C). Due to the lack of predicted transcription factors associated with rs2327429, which is located in the promoter region of TCF21, the four remaining sites were considered for inclusion in the study.

### Extraction and genotyping of DNA

Extraction of DNA was performed following our previous introductions [1]. The genotypes of the chosen tagSNPs were determined by the SNaPshot genotyping method, which has been validated in previous studies [21, 22]. Here are the primer sequences used (Table S1).

### Tissue samples and cells

A total of 120 pairs of tumorous and adjacent tissues were collected from radical gastric resection specimens of patients with GC in the First Affiliated Hospital of Nanjing Medical University. The study employed two human GC cell lines, MKN45 and AGS, obtained from the cell bank of the Chinese Academy of Sciences (Shanghai, China). The preservation of tissues and cell culture conditions were performed concerning a previous study [23]. All cells used in this study were authenticated using STR profiling within the last three years, and all experiments were performed with mycoplasma-free cells.

### **RNA extraction and quantitative real-time PCR** (**qRT-PCR**)

RNA was extracted from cells as well as 120 out of 890 pairs of tumorous tissues and adjacent normal tissues using TRIzol Reagent (Invitrogen, USA). Following reverse transcription, the expression of mRNA was measured by quantitative real-time PCR (ABI 7300). The primers used in this research are shown in the supplementary material. All experiments were performed in triplicate.

### Plasmid construction, siRNA interference, and transfection

We constructed luciferase reporter plasmids and TFAP2A-overexpressing plasmids in which the sequences contained the 2 kb upstream of the transcription start sites (TSS) of TCF21 with different alleles of rs2327430. The siRNA sequences designed against TFAP2A and TCF21 whose efficiency has been validated in other research are listed in the supplementary material [24, 25]. The rs2327430 wild (T) or mutant (C) vectors were obtained from GenePharma (GenePharma, Shanghai, China). When the cells were grown to 40–60% confluencein six-well plates, Lipofectamine 3000 (Invitrogen) and p3000 were used to transfect small interfering RNA (siRNA) and plasmids into the aforementioned cells.

### Western blotting

The protein extraction method and detailed steps of western blotting were adapted from a previous study [23]. The following antibodies were used: TCF21 and cleaved caspase 3 from Abcam (Britain) and p-AKT, AKT, caspase 3, BCL-xL, TFAP2A, and GAPDH from Proteintech (Wuhan, China).

### **Immunohistochemistry** (**IHC**)

After fixing and then embedding all specimens, we de-waxed the paraffin-embedded sections in xylene and incubated them with corresponding antibodies.

### Luciferase assay

In total, 0.4 µg luciferase reporter plasmids containing different alleles of rs2327430 were transfected into MKN45 and AGS cells, and equivalent amounts of TFAP2A-overexpressing plasmids were cotransfected for the luciferase assay. The empty vector served as a negative control. The luciferase assay was performed on the Dual-Luciferase Reporter Assay System (Promega, USA), and the outcomes were evaluated based on firefly luciferase activities normalized to Renilla luciferase.

### **Chromatin immunoprecipitation** (**ChIP**)

For the ChIP assay, we employed a ChIP assay kit (Beyotime, China). Broadly, we sonicated the cross-linked chromatin DNA into 200–1000 bp fragments that were immunoprecipitated with an anti-TFAP2A antibody (Proteintech, China), while normal rabbit IgG was used as the reference group. Quantification of the immunoprecipitated DNA was assessed by qRT-PCR with SYBR Green Mix (Vazyme Biotech Co., Ltd, China), and the results are presented relative to input (% input). The TCF21 promoter primers used are as follows: forward 5′-AGATGGACAGAACATGCTGC-3′ and reverse 5′-AGGGAAACTCAATGCACAGA CC-3′.

### Cell proliferation, wound healing, and transwell assays

As described in our previous study [23], cell counting kit 8 (CCK-8) and colony formation assays were performed to validate the influence of TFAP2A on the proliferative capacity of pre-treated GC cells altered by TCF21, while transwell assays and wound healing assays were used to investigate the effect on cell migration and invasion. All the above experiments were conducted in triplicate.

### Flow cytometric analysis of apoptosis

We conducted the apoptosis assay using an Annexin V-APC/PI Apoptosis Detection Kit (Multisciences, China). The obtained data were analyzed with flow cytometry (BD Biosciences, USA). The total apoptosis rate was calculated based on the ratio of early and late apoptotic cells.

### Animal experiment

To construct the tumor xenograft model, a total of 30 female nude mice were randomly assigned. Pre-treated cells (1 × 10^6^ cells/100 µl of PBS) were injected into the flank of each nude mouse (BALB/c) in the different groups, respectively. We measured tumor volume every 2 days and weighed xenografts after sacrificing the mice.

### False-positive report probability

According to previously reported studies [26], false-positive report probability (FPRP) was provided, and a false-positive rate between genetic polymorphisms and GC hazard was available (Table S2).

### Statistical analysis

SPSS22.0 software (SPSS Inc., Chicago, IL, USA) was chosen for performing the statistical analysis in our research. Statistical methods were used as described in our previous studies [1].

## Results

### Demographic information of the study subjects

The baseline characteristics of the 890 cases and 890 cancer-free controls are summarized in Table 1. These characteristics were well-matched between the two groups (*P* > 0.05). Among the GC patients, there were 490 cases of non-cardia cancer (55.1%). Cases with lymph node metastasis accounted for a greater proportion (62.0%), while a lower proportion had lymphovascular invasion (39.6%) and perineural invasion (39.6%). The distributions of the depth of invasion are presented below: 24.7% (T1), 11.8% (T2), 25.5% (T3), and 38.0% (T4). By the 8th edition of the AJCC cancer staging manual, the TNM stages of 890 patients were categorized as follows: stage I (27.9%), stage II (23.9%), stage III (42.7%), and stage IV (5.5%).

**Table 1:** Demographic information

Characteristics	Cases (*n* = 890)	Controls (*n* = 890)	*P* value
Age (years, mean ± SD)	61.48 ± 9. 347	60.55 ± 14.686	0.112
Sex [*n* (*%*)]			
Female	257 (29.9)	295 (33.1)	
Male	633 (71.1)	595 (66.9)	0.058
Hypertension [*n* (*%*)]			
No	592 (66.5)	568 (63.8)	
Yes	298 (33.5)	322 (36.2)	0.253
Diabetes [*n* (*%*)]			
No	781 (87.8)	776 (87.2)	
Yes	109 (12.2)	114 (12.8)	0.775
Smoking status [*n* (*%*)]			
No	671 (75.4)	706 (79.3)	
Yes	219 (24.6)	184 (20.7)	0.054
Drinking status [*n* (*%*)]			
No	729 (81.9)	754 (84.7)	
Yes	161 (18.1)	136 (15.3)	0.127
Residence [*n* (*%*)]			
Rural	489 (54.9)	479 (53.8)	
Urban	401 (45.1)	411 (46.2)	0.476
Tumor size [*n* (*%*)]			
<4 cm	625 (70.2)		
≥4 cm	265 (29.8)		
Tumor site [*n* (*%*)]			
Cardia	400 (44.9)		
Non-cardia	490 (55.1)		
Tumor differentiation [*n* (*%*)]			
Well + moderate	183 (20.6)		
Poor	707 (79.4)		
Depth of invasion [*n* (*%*)]			
T1	220 (24.7)		
T2	105 (11.8)		
T3	227 (25.5)		
T4	338 (38.0)		
LNM [*n* (*%*)]1			
N0	338 (38.0)		
N1	121 (13.6)		
N2	133 (14.9)		
N3	298 (33.5)		
TNM stage [*n* (*%*)]			
I	248 (27.9)		
II	213 (23.9)		
III	380 (42.7)		
IV	49 (5.5)		
LVI [*n* (*%*)]			
No	538 (60.4)		
Yes	352 (39.6)		
PNI [*n* (*%*)]			
No	538 (60.4)		
Yes	352 (39.6)		
Lauren classification ^a^ [*n* (*%*)]			
Intestinal	276 (40.5)		
Diffuse	180 (26.4)		
Mixed	225 (33.0)		

*SD* standard deviation, *LNM* Lymph node metastasis, *LVI* lymphovascular invasion, *PNI* perineural invasion

^a^ The information was not recorded in 209 GC patients

### Associations between TCF21 polymorphisms and the risk of GC

Due to the absence of allele C (*n* = 0) in the genotyping results, rs3734281 was not investigated. Table 2 shows the frequencies of genotypes and alleles at each selected SNP locus. However, the examination of HWE showed disequilibrium in rs12190287 (Table S3); therefore, further analysis was not conducted for this SNP. The results of rs2327430 and rs4896011 were the focus of our study. As presented in Table 2, the presence of the C allele of rs2327430 could decrease the risk of GC occurrence (OR = 0.78, 95% CI = 0.63–0.97), which was also observed in the dominant model (OR = 0.74, 95% CI = 0.58–0.93). In addition, the TC genotype in the codominant model played an active role in reducing the risk of GC (OR = 0.72, 95% CI = 0.56–0.92). Conversely, the A allele in rs4896011 was associated with an increased risk of GC in the codominant mode (OR = 1.36, 95% CI = 1.07–1.75) as well as the dominant model (OR = 1.39, 95% CI = 1.09–1.77).

**Table 2:** The association between TCF21 gene polymorphisms (rs2327430, rs4896011) and the risk of gastric cancer

Genotype	Cases *n*	Controls *n*	OR (95% CI)	*P* value	OR (95% CI)^a^	*P* value
Overall	890	890				
rs2327430						
Additive model			**0.78** (**0.62–0.97**)	**0.024**	**0.77** (**0.62–0.97**)	**0.023**
Codominant model						
TT	739	697	1		1	
TC	143	187	**0.72** (**0.57–0.92**)	**0.008**	**0.72** (**0.56–0.92**)	**0.008**
CC	8	6	1.26 (0.43-3. 64)	0.673	1.26 (0.43–3.66)	0.676
Dominant model						
TT	739	697	1		1	
TC + CC	151	193	**0.74** (**0.58–0.94**)	**0.012**	**0.74** (**0.58–0.93**)	**0.011**
Recessive model						
TT + TC	882	884	1		1	
CC	8	6	1.34 (0.46–3.87)	0.593	1.34 (0.46–3.89)	0.596
Allele						
T	1621	1581	1			
C	159	199	**0.78** (**0.63–0.97**)	**0.026**		
HWE		0.084				
rs4896011						
Additive model			**1.41** (**1.12–1.79**)	**0.004**	**1.39** (**1.10–1.77**)	**0.006**
Codominant model						
TT	706	751	1		1	
TA	178	137	**1.38** (**1.08–1.77**)	**0.01**	**1.36** (**1.07–1.75**)	**0.014**
AA	6	2	3.19 (0.64–15.86)	0.156	3.07 (0.61–15.38)	0.172
Dominant model						
TT	706	751	1		1	
TA + AA	184	139	**1.41** (**1.10–1.80**)	**0.004**	**1.39** (**1.09–1.77**)	**0.009**
Recessive model						
TT + TA	884	888	1		1	
AA	6	2	3.01 (0.61–14.97)	0.177	2.89 (0.58–14.48)	0.196
Allele						
T	1590	1639	1			
A	190	141	**1.39** (**1.11–1.75**)	**0.005**		
HWE		0.099				

*OR* odds ratio, *CI* confidence interval, *HWE* Hardy–Weinberg expectations

^a^Adjusted for age, sex, smoking status, drinking status, residence, hypertension, and diabetes in the logistic regression model

The significant results are in bold

### Stratification analysis of TCF21 SNPs and GC risk

To clarify the potential effects of risk factors and comorbidities related to GC, a subgroup analysis was conducted using dominant models of rs2327430 and rs4896011. Table 3 presents the results for rs2327430, indicating that the protective effects of mutant genotypes TC + CC were particularly significant among females (OR = 0.52, 95% CI = 0.33–0.82) and patients residing in urban areas (OR = 0.67, 95% CI = 0.47–0.95), without hypertension (OR = 0.67, 95% CI = 0.50–0.90), without diabetes (OR = 0.76, 95% CI = 0.59–0.98), without smoking history (OR = 0.68, 95% CI = 0.56–0.94), and without drinking history (OR = 0.73, 95% CI = 0.56–0.95). For rs4896011, the AT + AA variant genotypes were found to significantly increase the risk of GC in males (OR = 1.56, 95% CI = 1.17–2.08), individuals without hypertension (OR = 1.67, 95% CI = 1.22–2.29), individuals without diabetes (OR = 1.40, 95% CI = 1.07–1.83), nonsmokers (OR = 1.48, 95% CI = 1.12–1.97), nondrinkers (OR = 1.40, 95% CI = 1.07–1.84), and individuals living in rural areas (OR = 1.40, 95% CI = 1.00-1.97). No obvious correlations were observed in the opposite subgroups.

**Table 3:** Stratified analysis for rs2327430 and rs4896011 genotype in cases and controls

Variables	*n* CT + CC / *n* TT for rs2327430		Logistic regression for rs2327430		*n* AT + AA / *n* TT for rs4896011		Logistic regression for rs4896011	
	Cases *n* = 890	Controls *n* = 890	Adjusted OR (95% CI) ^a^	*P* value	Cases *n* = 890	Controls *n* = 890	Adjusted OR (95% CI) ^a^	*P* value
Age(years)								
≥ 60	94/443	110/398	0.75 (0.55–1.03)	0.075	112/425	80/428	1.36 (0.99–1.88)	0.059
<60	57/296	83/299	0.69 (0.48–1.01)	0.057	72/281	59/323	1.34 (0.91–1.98)	0.137
Gender								
Males	115/518	127/468	0.82 (0.62–1.09)	0.169	146/487	97/498	**1.56** (**1.17–2.08**)	**0.003**
Females	36/221	66/229	**0.52** (**0.33–0.82**)	**0.005**	38/219	42/253	1.07 (0.66–1.75)	0.786
Hypertension								
No	98/494	128/440	**0.67** (**0.50–0.90**)	**0.007**	129/463	78/490	**1.67** (**1.22–2.29**)	**0.001**
Yes	53/245	65/257	0.85 (0.56–1.27)	0.425	55/243	61/261	0.94 (0.62–1.42)	0.768
Diabetes								
No	132/649	164/612	**0.76** (**0.59–0.98**)	**0.035**	156/625	115/661	**1.40** (**1.07–1.83**)	**0.013**
Yes	19/90	29/85	0.59 (0.30–1.16)	0.128	28/81	24/90	1.23 (0.65–2.33)	0.531
Smoking status								
No	113/558	160/546	**0.68** (**0.56–0.94**)	**0.006**	137/534	103/603	**1.48** (**1.12–1.97**)	**0.006**
Yes	38/181	33/151	0.78 (0.43–1.41)	0.409	47/172	36/148	1.18 (0.71–1.95)	0.522
Drinking status								
No	122/607	163/591	**0.73** (**0.56–0.95**)	**0.016**	143/586	111/643	**1.40** (**1.07–1.84**)	**0.016**
Yes	29/132	30/106	0.78 (0.43–1.40)	0.401	41/120	28/108	1.41 (0.80–2.47)	0.237
Residence								
Rural	83/409	97/382	0.78 (0.56–1.08)	0.135	101/388	72/407	**1.40** (**1.00-1.97**)	**0.049**
Urban	68/333	96/315	**0.67** (**0.47–0.95**)	**0.023**	83/318	67/344	1.35 (0.94–1.94)	0.1

*OR* odds ratio, *CI* confidence interval

^a^Adjusted for age, sex, smoking status, drinking status, residence, hypertension, and diabetes (excluding the stratifed factor in each stratum) in the logistic regression model

The significant results are in bold

Further analyses were conducted to assess the correlations between the aforementioned tagSNPs and various clinicopathological characteristics. However, obvious proof of relevance between them was rarely obtained (Table S4).

### Associations of TCF21 polymorphisms with clinicopathological characteristics and the prognosis of GC patients

**Fig. 1:**
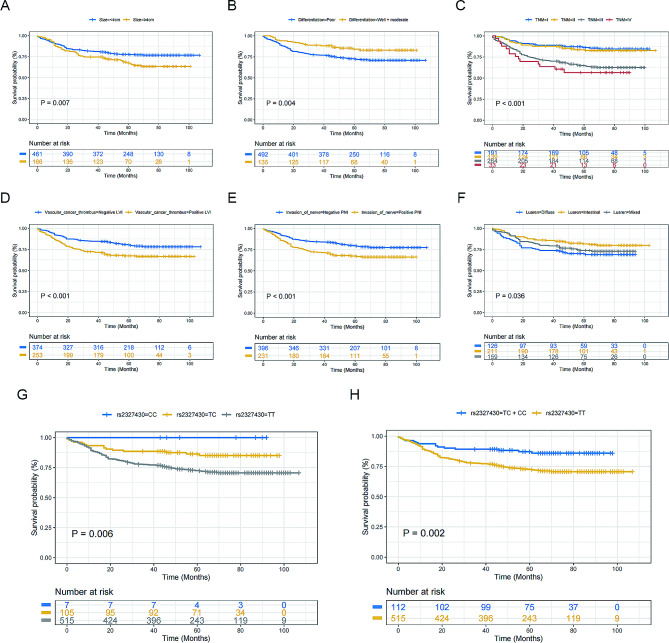
The associations of various factors with the prognosis of GC patients. *Notes*: (**A**–**F**) Kaplan-Meier survival curves for the overall survival by characteristics and clinical features of gastric cancer patients. (tumor size, tumor differentiation, TMN stage, LVI, PNI, Lauren classification.) (**G** and **H**) Kaplan-Meier survival curves of rs2327430 polymorphism for the overall survival in patients with gastric cancer. (TT vs. TC, TT vs. CC, TT vs. TC + CC.)

Analysis of the correlation between the clinicopathological features and the prognosis of GC patients indicated that larger tumor size, positive lymphovascular invasion, more advanced TNM stage (III and IV), and the diffuse type based on Lauren classification were independent predictors for poor outcome of GC patients (Table 4) (Fig. 1A-F), which aligns with their recognized roles. Based on the preliminary findings of this study, we explored the potential effects of the two aforementioned SNPs on the overall survival of GC patients. Unfortunately, analyses between wild-type homozygotes and mutant homozygotes (with SNPs affecting both alleles) could not be performed, and it was not possible to make predictions based on the MST data due to the limitation of the small sample size and single survival outcome of mutant homozygote samples. According to the results obtained thus far, the TC genotype (HR = 0.50, 95% CI = 0.27–0.91) and TC + CC genotype (HR = 0.47, 95% CI = 0.26–0.85) in rs2327430 could have a favorable impact on prognosis (Fig. 1G-H), while the polymorphisms in rs4896011 did not correlate with overall survival in GC patients when tumor size, lymphovascular invasion, TNM stage, and Lauren classification were considered (Table 5).

**Table 4:** The association of patients’ clinical features and overall survival time

Variables	Patients	Deaths	MST (months)	Log-rank *P*	HR (95%CI)	HR (95% CI) ^a^
Overall^‡^	627	158				
Age (years)						
<60	249	54	88		1	1
≥60	378	104	79.6	0.085	1.33 (0.96–1.85)	1.31 (0.94–1.84)
Sex						
Male	452	117	84.2		1	1
Female	175	41	81.4	0.592	0.91 (0.64–1.30)	0.91 (0.64–1.30)
Tumor size						
<4 cm	461	103	86.9		1	1
≥4 cm	166	55	74.2	**0.007**	**1.56** (**1.12–2.16**)	**1.51** (**1.09–2.10**)
Tumor site						
Cardia	295	67	86.8		1	1
Non-cardia	332	91	79.9	0.2	1.23 (0.90–1.68)	1.25 (0.91–1.72)
Tumor differentiation						
Well + moderate	135	21	88.5		1	1
Poor	492	137	82.2	**0.004**	**1.92** (**1.21–3.04**)	1.26 (0.77–2.05)
Depth of invasion						
T1	166	23	90.5		1	1
T2	83	14	88.9		1.23(0.63–2.38)	1.18(0.60–2.29)
T3	158	47	80.9		**2.35** (**1.43–3.87**)	**2.31** (**1.40–3.80**)
T4	220	74	72.3	**< 0.001**	**2.74** (**1.71–4.37**)	**2.66** (**1.67–4.26**)
LNM^§^						
N0	239	35	94.2		1	1
N1	90	16	85.6		1.25 (0.69–2.26)	1.20 (0.66–2.17)
N2	84	22	79.4		**1.93** (**1.13–3.28**)	**1.88** (**1.10–3.21**)
N3	214	85	67.4	**< 0.001**	**3.21** (**2.17–4.77**)	**3.26** (**2.20–4.84**)
TNM stage						
I	191	27	91.1		1	1
II	139	23	92.6		1.17 (0.67–2.04)	1.14 (0.65–1.99)
III	264	94	70.8		**2.88** (**1.88–4.42**)	**2.84** (**1.85–4.36**)
IV	33	14	58.9	**< 0.001**	**3.67** (**1.92−7.00**)	**3.71** (**1.94–7.08**)
LVI						
No	374	76	89.4		1	1
Yes	253	82	75.1	**< 0.001**	**1.79** (**1.31–2.45**)	**1.42** (**1.03–1.96**)
PNI						
No	396	82	88.7		1	1
Yes	231	76	72.9	**< 0.001**	**1.73** (**1.26–2.36**)	1.23 (0.89–1.71)
Lauren classification ^¶¶^						
Intestinal	211	39	87.4		1	1
Diffuse	126	38	70.4		**1.77** (**1.13–2.77**)	**1.63** (**1.03–2.57**)
Mixed	159	41	75	**0.036**	1.44 (0.93–2.23)	1.24 (0.80–1.92)

*MST* median survival time; *HR* hazard ratio; *CI* confidence interval; *LNM* Lymph node metastasis; *LVI* lymphovascular invasion; *PNI* perineural invasion

^‡^The information was not available in 263 patients

^a^ Adjusted by age, gender, and TNM stage in Cox regression multivariate analysis

^¶^ The information was not recorded in 165 GC patients

The significant results are in bold

**Table 5:** Associations between rs2327430 and rs4896011 genotypes and GC overall survival in 627 GC patients

Genotype	All cases				Univariate	*P*	Multivariate	*P* ^a^
	Patients	Deaths	MST ^b^	Log-rank *P*	HR (95% CI)		HR (95% CI) ^a^	
rs2327430								
TT	515	143			1.00		1.00	
CT	105	15		**0.006**	**0.47** (**0.28–0.80**)	**0.006**	**0.50** (**0.27–0.91**)	**0.022**
TT	515	143	82.4					
CT + CC	112	15	87.2	**0.002**	**0.44** (**0.26–0.75**)	**0.003**	**0.47** (**0.26–0.85**)	**0.012**
rs4896011								
TT	493	127			1.00		1.00	
AT	130	31		0.491	0.90 (0.61–1.34)	0.606	1.00 (0.64–1.57)	0.992
TT	493	127	84.1		1.00		1.00	
AT + AA	134	31	81.5	0.493	0.87 (0.59–1.29)	0.495	0.96 (0.61–1.51)	0.859

*MST* median survival time, *HR* hazard ratio, *CI* confidence interval

^a^ Adjusted for Tumor size, TNM stage, LVI, and Lauren classifIcation in Cox regression multivariate analysis

^b^ Limited by small sample size of the homozygote mutation, part of the data could not be calculated

The significant results are in bold

#### The C allele of rs2327430 modulates malignant behaviors in GC cells

**Fig. 2:**
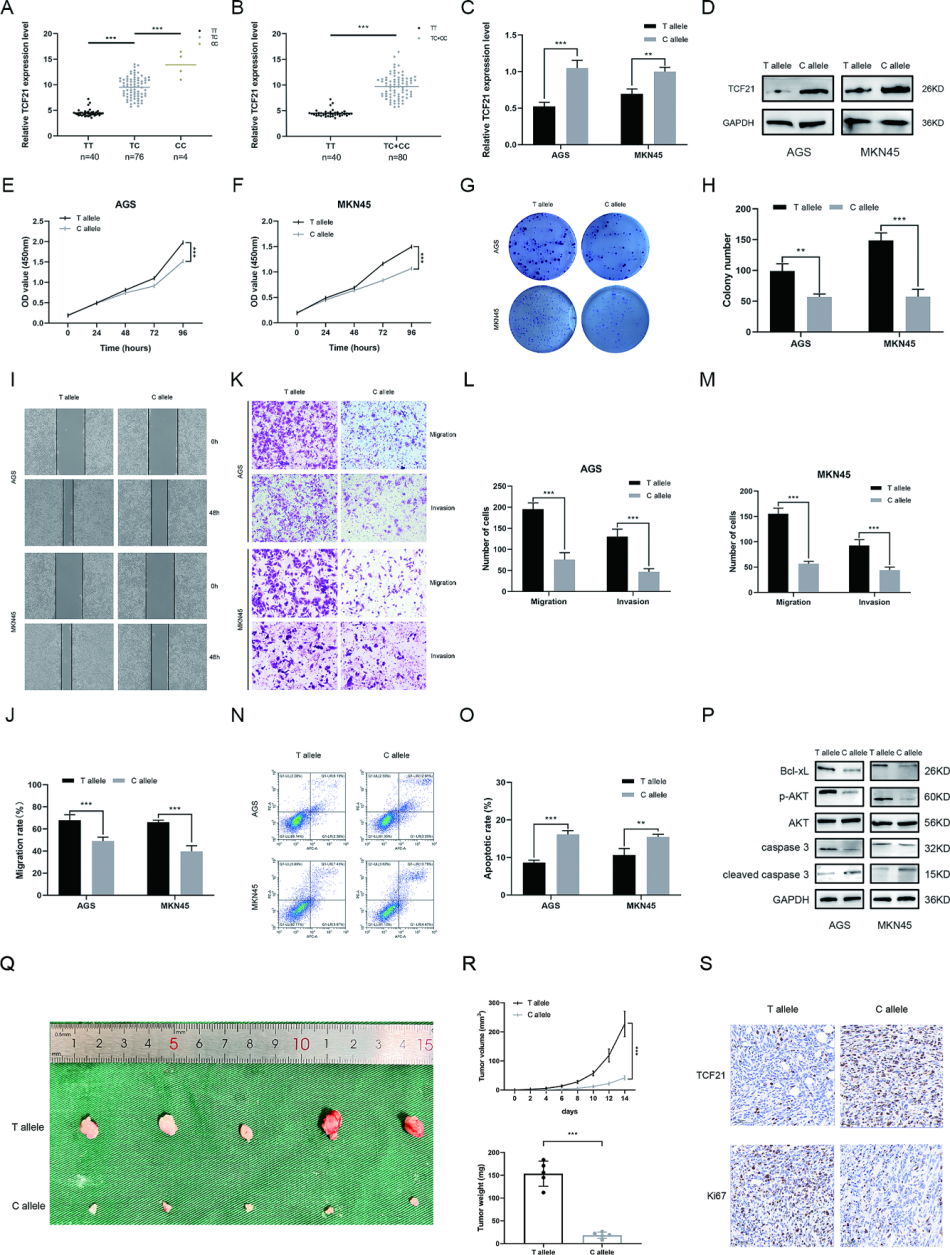
The C allele in rs232730 inhibited the malignant behavior of GC cell lines relative to the T allele by affecting the expression level of TCF21 *Notes*: (**A** and **B**) Correlation between different rs2327430 genotypes and TCF21 expression levels in tumor tissues. (**C** and **D**) The expression level of TCF21 was analyzed in GC cell lines transfected with different alleles of rs2327430 by qRT-PCR and western blots. (**E–H**) CCK-8 and colony formation assay showed the C allele in rs2327430 could inhibit the proliferation ability of GC cell lines. (**I–M**) The wound healing assay and transwell assay were performed to show the effect of rs2327430 on the migration and invasion of GC cell lines. (**N** and **O**) The apoptotic rate of GC cell lines transfected with different alleles in rs2327430 was examined by flow cytometry. (**P**) The effect of different alleles in rs2327430 on the AKT/Bcl-xL signaling pathway was examined by western blots. (**Q**) Images of the subcutaneous xenografts were presented. (**R**) tumor growth curves were drawn and the tumor weight was calculated. (**S**) IHC was used to examine the expression of TCF21 and Ki67 in xenografts

Given the protective roles of rs2327430 in both the risk and prognosis of GC, we further explored the underlying mechanism. Bioinformatic analyses showed a high abundance of promoter and enhancer histone marks in the 2000 bp upstream genomic region of TCF21, especially around rs2327430 (Fig. S1A-B). The impact of rs2327430 on the open chromatin state was also investigated by single-cell assay for transposase-accessible chromatin using sequencing (scATAC-seq) (Fig. S1D). The 3DSNP database and UCSC database were utilized to visualize the structure of rs2327430 through circular and linear plots, respectively (Fig. S1E-F). In addition, we predicted the role of rs2327430 in modulating RNA secondary structure (Fig. S1G). Preliminary exploratory analysis indicated that rs2327430 may regulate promoter activity by altering the binding of a specific transcription factor. First, we quantified the expression of TCF21 in 120 GC tissues by qRT-PCR. The results indicated that cases carrying the rs2327430 C allele had a higher expression level of TCF21 than those with the TT genotype (Fig. 2A-B). Based on the above observations, we examined the expression level of TCF21 in cells transfected with plasmids carrying different alleles of rs2327430, and the rs2327430 C allele caused an increase in TCF21 expression at both the RNA level and protein level (Fig. 2C-D). The CCK-8 assay and colony formation assay validated that the C allele could inhibit the proliferation of GC cells (Fig. 2E-H). The migration and invasion capacity of GC cells may be weakened by the C allele of rs2327430 (Fig. 2I-M). The C allele may also be associated with a higher apoptosis rate in GC cells than the T allele (Fig. 2N-O). A previous study validated that TCF21 plays a role in GC through the AKT-Bcl-xL signaling pathway [15]. As a result, we explored whether there was a correlation between rs2327430 and the AKT-Bcl-xL signaling pathway, and we obtained a positive result (Fig. 2P).

In vivo, subcutaneous xenograft tumor models were constructed, and the expression of TCF21 as well as Ki67 was examined by IHC, whose results validated the aforementioned conclusion as well (Fig. 2Q-S).

### The C allele in rs2327430 blocks the binding of TFAP2A to the TCF21 promoter

**Fig. 3:**
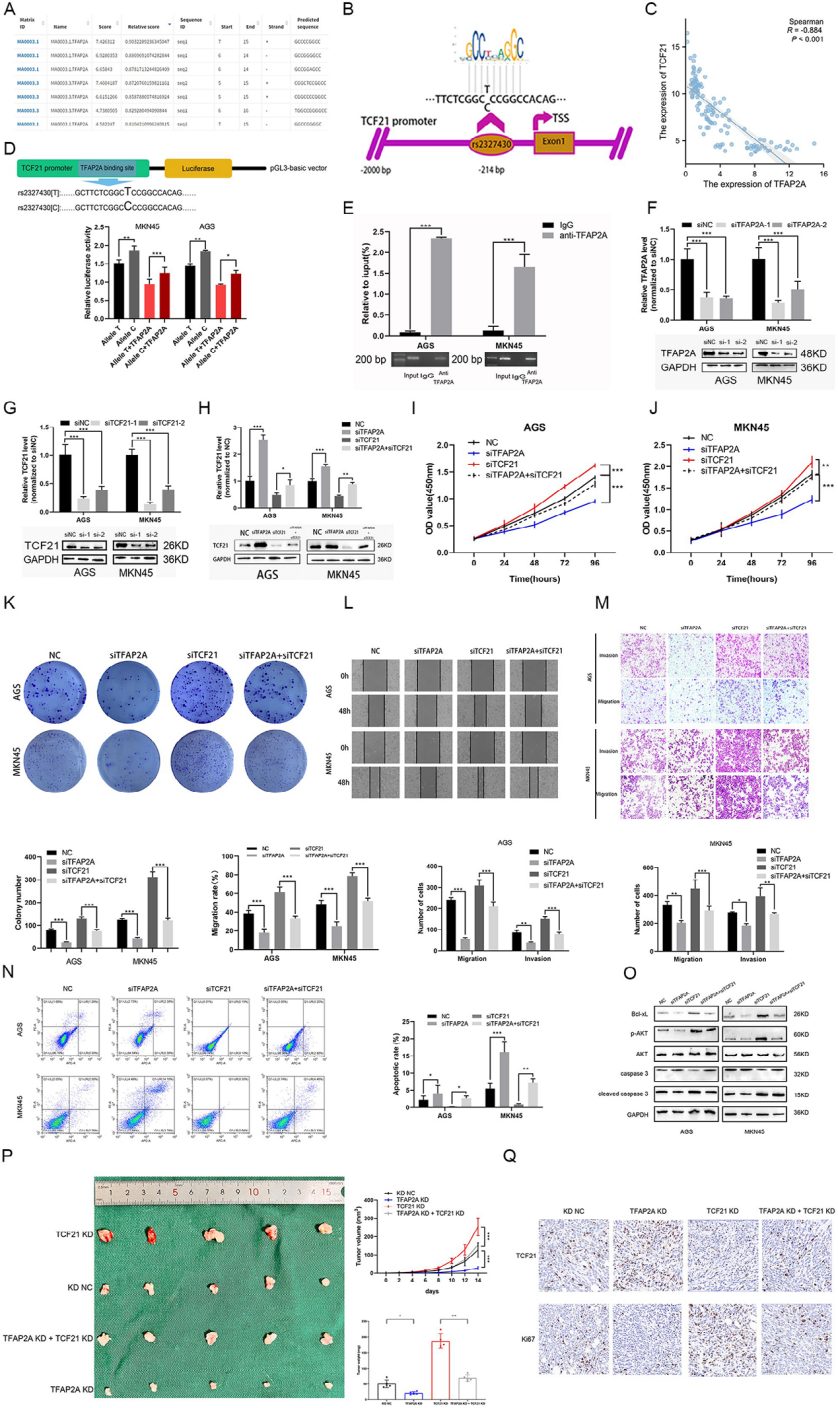
The C allele of rs2327430 played a role in GC by blocking the binding of TFAP2A to the TCF21 promoter. *Notes*: (**A** and **B**) The potential of the binding between tagSNPs **and** transcription factor was analyzed and displayed according to the JASPAR database. (**C**) Correlation analysis between TFAP2A and TCF21 mRNA was performed in 120 GC patients and analyzed with Spearman’s correlation test. (**D**) Schematic illustration of the reporter gene containing rs2327430 C or T allele constructs and reporter plasmids with the different alleles of rs2327430 were transfected into AGS and MKN45 cells. Then, the results were expressed as relative luciferase activity (Firefly luciferase/Renilla luciferase). Both cells were also co-transfected with TFAP2A plasmid, respectively. (**E**) CHIP assay evaluated by qRT-PCR was performed with control IgG or antibody against TFAP2A in the AGS and MKN45 cells, results of which are normalized to the input group and shown as means ± SEM in 3 independent experiments. (**F** and **G**) The knock-down efficiency of siRNAs on TCF21 and TFAP2A was determined by qRT-PCR and western blots, respectively. (**H**) The rescue experiment was carried out to verify whether the expression of TCF21 is affected by TFAP2A through qRT-PCR and western blot. (**I–N**) It is shown that TCF21 knockdown could partially rescue functional phenotypes caused by TFAP2A knockdown including proliferation, invasion, metastasis, and apoptosis. (**O**) The effect of TFAP2A knockdown on AKT/Bcl-xL signaling pathway could be rescued partially by TCF21 knockdown. (**P**) Images of the subcutaneous xenografts were presented. tumor growth curves were drawn and the tumor weight was calculated. (**Q**) IHC was used to examine the expression of TCF21 and Ki67 in xenografts

**Fig. 4:**
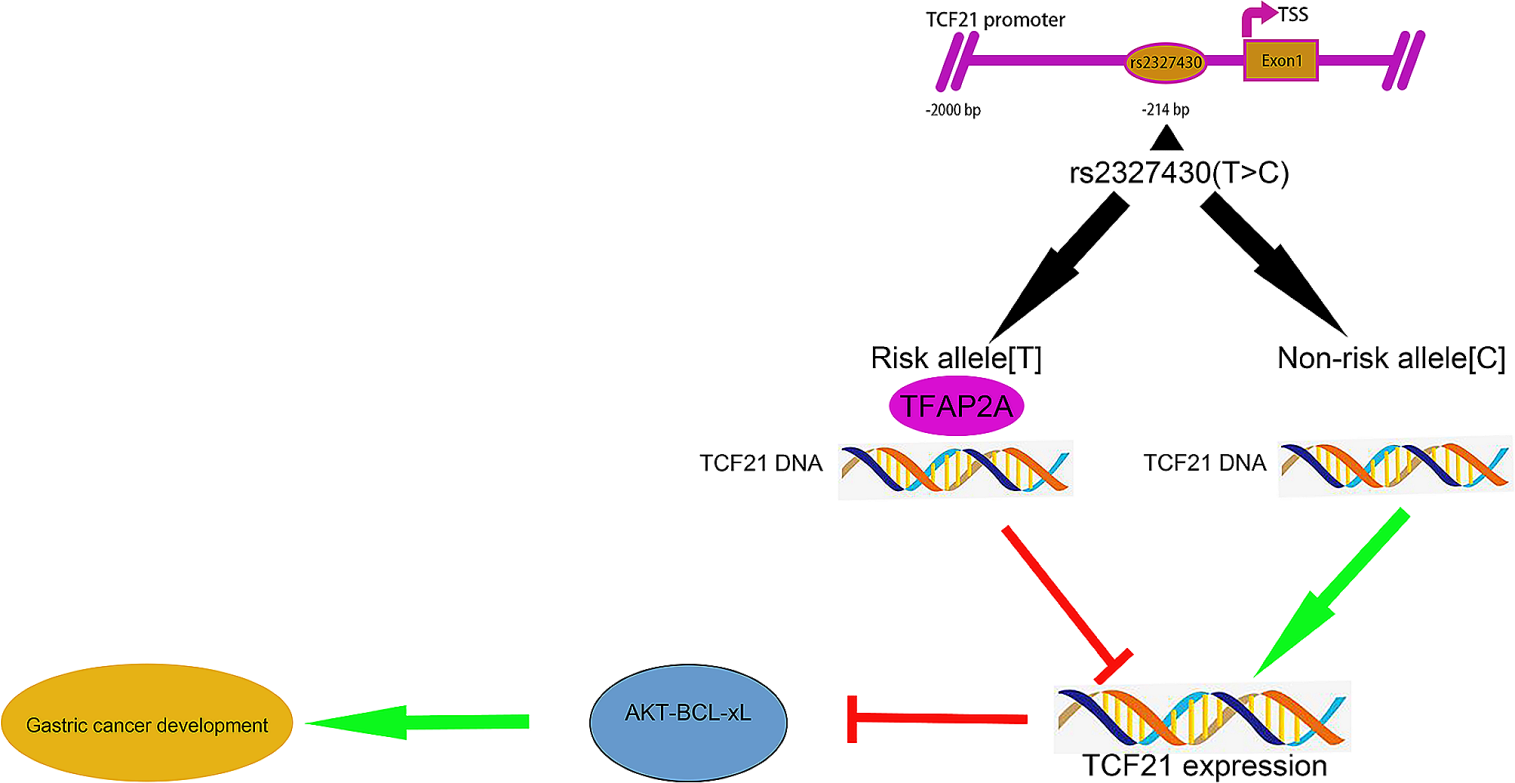
The role of rs2327430 in modulating gastric cancer. *Notes*: The allele C played a protective role in gastric cancer development by inhibiting the binding with TFAP2A

To understand the underlying mechanisms, we utilized the JASPAR database and found that there existed a TFAP2A binding motif in rs2327430 (Fig. 3A-B). Analysis of molecular correlates also showed that the expression level of TCF21 was negatively correlated with the expression level of TFAP2A (Fig. 3C). To further validate this conjecture, various types of luciferase reporter vectors with promoters containing either the rs2327430 allele (T or C) were constructed and transfected into MKN45 (rs2327430-TT) and AGS (rs2327430-TC) cells for the luciferase assay. Additionally, a plasmid overexpressing TFAP2A was cotransfected into these cells. The cells transfected with a vector carrying the C allele exhibited significantly increased luciferase activity compared to those with the T allele, and the overexpression of TFAP2A negatively affected luciferase activity (Fig. 3D). The study revealed a negative correlation between the expression levels of TFAP2A and the transcriptional activity of TCF21. Furthermore, it was observed that the T allele inhibited the transcription of TCF21. The direct binding of TFAP2A to rs2327430 in TCF21 was confirmed by ChIP allele-specific qPCR assay in the two cell lines (Fig. 3E). To perform the following rescue assays, the efficiency of siRNAs in knockdown of TCF21 and TFAP2A was determined by qRT‒PCR and western blots, respectively (Fig. 3F-G). TFAP2A promotes malignant cell behaviors in GC, while TCF21 plays a protective role in GC [15, 25]. To further substantiate these findings, rescue experiments were conducted in vitro. Quantitative qRT-PCR and western blotting were employed to validate the impact of TFAP2A on the expression of TCF21 (Fig. 3H). In accordance with the above results, the knockdown of TFAP2A partially rescued several functional phenotypes, such as proliferation, migration, invasion, and apoptosis when TCF21 was also knocked down (Fig. 3I-O). A similar phenomenon was also observed in vivo experiments (Fig. 3P-Q). In summary, the polymorphisms in rs2327430 influenced malignant behaviors and the overall survival of GC patients by altering the binding with TFAP2A (Fig. 4).

## Discussion

SNPs are considered to be closely related to environmental, ethnic, and hereditary factors [27]. Specific SNP sites could result in changes in gene expression profiles and gene functions, thereby influencing susceptibilities to various diseases, including tumors [28, 29]. Genome-wide association studies (GWASs) and high-throughput sequencing have identified numerous unstudied SNP sites, providing new perspectives on the mechanisms of tumorigenesis and methods for cancer screening [30, 31]. Additionally, there have been publications linking genetic polymorphisms of TCF21 to numerous diseases [16, 32]. Considering the above background, we focused on the association between polymorphisms of TCF21 and GC. After conducting statistical analyses, allele C in rs2327430 was found to be associated with a lower incidence of GC, and the TC + CC genotype was associated with a better prognosis.

As reported, SNPs occurring at any location in the genome have the potential to influence the trait. For instance, in the coding DNA sequence (CDS) region, nonsynonymous mutations could alter the amino acid encoded by influencing codons, while synonymous mutations may affect RNA secondary structures, protein folding, and cellular localization. Mutations located in UTRs play a role in regulating the expression of related genes, highlighting the significant implications of studying SNP sites in different parts of the genome [33–35]. SNP positioning in promoter regions can directly modulate gene transcriptional activity, attracting considerable attention from researchers [36, 37]. We took notice of the effects on binding with a transcription factor, mainly because rs2327430 is positioned 214 bp upstream of the first exon of TCF21. The conclusion that TFAP2A may act as a transcriptional repressor in this process was tentatively proposed by us. Although little is known about the mechanism by which transcriptional repressors exert their effects, while much is known about transcriptional activators, numerous studies have revealed the function of allele-specific transcriptional repressor binding sites [32, 38–40], which provided a theoretical basis for this study.

During the statistical analysis, the association of the C allele in rs2327430 with GC seemed to be driven by the heterozygous genotype, whereas the homozygous CC genotype had an OR of 1.26. However, the sample size of patients with the homozygous CC genotype may have resulted in a discrepancy between the direction of the correlations for the heterozygous TC and homozygous CC genotypes. The observation of a rare association between polymorphisms and clinicopathological features in the stratified analysis was noteworthy. Interestingly, one study recently reported a similar phenomenon [41]. This phenomenon may be attributed to many factors in vivo that differ from those in vitro. First, TCF21 is part of a complex regulatory network, while tumors are regulated by multiple molecules and axes. Additionally, the heterogeneity of the tumor may contribute to the limited impact of SNPs on clinicopathological characteristics at the population level. A larger sample size and a wider population range may be beneficial to reducing the effect of individual differences. Furthermore, strong immune-mediated surveillance and clearance could play an unpredictable role in vivo [42, 43]. Moreover, the interindividual variation in tumor metabolism and the complex tumor microenvironment in vivo may weaken the efficacy of SNPs [44, 45]. The protective effects on GC patients’ overall survival may indicate that these SNPs not only inhibit malignant properties but also alter the subsequent chemoradiotherapy sensitivity of GC [46, 47].

The mechanism by which TCF21 affects malignant behaviors in GC is also noteworthy. TCF21 has been reported to influence multiple pathways, including the PI3K-AKT pathway and MAPK pathway [48, 49]. However, further validation is needed to identify the direct target genes of TCF21 that regulate these downstream signaling pathways. This validation is of great value for future research.

Despite the significant discoveries revealed by our study, we must acknowledge its limitations. Above all, the modes by which transcriptional repressors work are diverse; some compete against transcriptional activators, and others may mediate chemical modifications. However, the mechanism of the transcriptional inhibition of TCF21 exerted by TFAP2A awaits further exploration [50]. Moreover, the reason that rs4896011 is associated with the occurrence but not the development of GC is still unclear. In addition, *H. pylori* infection, one of the major risk factors for GC in China, was not included in our study due to a lack of relevant medical records for a significant number of patients. Finally, the estimates of MST and the analysis of SNP correlation with clinicopathological data were imperfect due to limitations imposed by the small sample size and missing pathological information and survival results.

### Electronic supplementary material

Below is the link to the electronic supplementary material.

Supplementary Material 1

## Data Availability

No datasets were generated or analysed during the current study.
