# Supplementary material for: Polymorphism rs2327430 in TCF21 predicts the risk and prognosis of gastric cancer by affecting the binding between TFAP2A and TCF21
Source: Cancer Cell Int. 2024 May 7;24:159. doi: 10.1186/s12935-024-03343-z (PMC11075239; doi:10.1186/s12935-024-03343-z)
Supplement: Supplementary file 1 — Supplementary Material 1 [file 12935_2024_3343_MOESM1_ESM.pdf]

Supplementary material

Table S1 Primers used for PCR

Number †	SNPs for TCF21	Upstream primers	Downstream primers	Length of PCR product;GC content	Polymorphisms	Extended primers
S1	rs12190287	CGCTTCATTTGCTCTTTCTTT	CGTGAGTGGGAGATTGGAAA	556;51.6%	[C/G]	TGCAAATAGACAGGTGGATGAA
S2	rs2327430	ATTCGTGTTTTCTTGGGAGGA	GCCGTAGAGGAGGGTGAGC	407;51.4%	[C/T]	TTTTTTTTTTACAGAACATGCTGCTTCTCGGC
S3	rs3734281	GAGCCCTTCTCCCATTCCA	TTTGGTTGTTCTCCTGGTTCTT	314;50.3%	[C/T]	TTTTTTTTTTTACCCTTCTGGGAAGACCGGAG
S4	rs4896011	CGCTTCATTTGCTCTTTCTTT	CGTGAGTGGGAGATTGGAAA	556;51.6%	[A/T]	AGTCCCCAGTGTGATTCAATTG

† Numbers (S1-S4) were added to identify corresponding selected SNP.

Table S2 False-positive report probability analyses for the significant findings (rs2327430, rs4896011)

Genotypes	Crude OR (95%CI)	P-value	Statistical power †	Prior probability				
				0.25	0.1	0.01	0.001	0.0001
rs2327430								
Additive model	0.78 (0.62-0.97)	0.024	0.917	**0.073**	**0.191**	0.721	0.963	0.996
Codominant model								
TC/TT	0.72 (0.57-0.92)	0.008	0.722	**0.032**	**0.091**	0.532	0.917	0.991
CC/TT	1.26 (0.73-3.64)	0.673	0.628	0.763	0.906	0.991	0.999	1.000
Dominant model								
TC + CC/TT	0.74 (0.58-0.94)	0.012	0.791	**0.044**	**0.120**	0.600	0.938	0.993
Allele								
C/T	0.78 (0.63-0.97)	0.026	0.922	**0.078**	0.202	0.736	0.966	0.996
rs4896011								
Additive model	1.41 (1.12-1.79)	0.004	0.674	**0.017**	**0.051**	0.370	0.856	0.983
Codominant model								
TA/TT	1.38 (1.08-1.77)	0.010	0.731	**0.039**	**0.110**	0.575	0.932	0.993
AA/TT	3.19 (0.64-15.86)	0.156	0.178	0.724	0.888	0.989	0.999	1.000
Dominant model								
TA + AA/TT	1.41 (1.10-1.80)	0.004	0.647	**0.018**	**0.053**	0.380	0.861	0.984
Allele								
A/T	1.39 (1.11-1.75)	0.005	0.740	**0.020**	**0.057**	0.401	0.871	0.985

The significant results are in bold.

† Statistical power was calculated using the number of observations.

Table S3 The association between TCF21 gene polymorphisms (rs12190287) and the risk of gastric cancer

Genotype	Cases *n*	Controls *n*	OR (95% CI)	*P* value	OR (95% CI) [a]	*P* value
Overall	890	890				
rs12190287						
Additive model			0.90 (0.79-1.04)	0.160	0.90 (0.78-1.04)	0.162
Codominant model						
CC	334	306	1.00		1.00	
CG	448	465	0.88 (0.72-1.08)	0.226	0.88 (0.72-1.07)	0.203
GG	108	119	0.83 (0.61-1.13)	0.233	0.84 (0.62-1.13)	0.247
Dominant model						
CC	334	306	1.00		1.00	
CG + GG	556	584	0.87 (0.72-1.06)	0.167	0.87 (0.71-1.06)	0.154
Recessive model						
CC + CG	782	771	1.00		1.00	
GG	108	119	0.90 (0.68-1.18)	0.435	0.90 (0.68-1.20)	0.475
Allele						
C	1116	1077	1.00			
G	664	703	1.10 (0.96-1.26)	0.179		
HWE		0.005				

OR odds ratio, *CI* confidence interval, *HWE* Hardy–Weinberg expectations

[a] Adjusted for age, gender, smoking status, drinking status, residence, hypertension, and diabetes in the logistic regression model.

Table S4 Associations between TCF21 genotypes and clinicopathological features of gastric cancer (rs2327430, rs4896011)

Variables	*n* CT + CC/*n* TT for rs2327430		Logistic regression for rs2327430		*n* AT + AA/*n* TT for rs4896011		Logistic regression for rs4896011	
	n CT + CC	*n* TT	OR (95% CI) [a]	*P* value	*n* AT + AA	*n* TT	OR (95% CI) [a]	*P* value
Tumor size	.	.	.	.	.	.	.	.
＜4 cm	97	531	1.00		128	505	1.00	
≥4 cm	54	208	1.41 (0.97-2.04)	0.070	56	201	1.06 (0.74-1.52)	0.750
Tumor site								
Cardia	67	333	1.00		84	316	1.00	
Non-cardia	84	406	3.02 (0.35-26.20)	0.317	100	390	0.22 (0.02-2.53)	0.225
Tumor differentiation								
Well + moderate	30	153	1.00		45	138	1.00	
Poor	121	586	1.07 (0.69-1.66)	0.770	139	568	0.74 (0.50-1.09)	0.152
Depth of invasion								
T1 + T2	63	262	1.00		72	253	1.00	
T3 + T4	88	477	0.76 (0.53-1.08)	0.125	112	453	0.83 (0.59-1.16)	0.272
LNM stage								
N0	54	284	1.00		73	265	1.00	
N1	21	100	1.10 (0.63-1.92)	0.734	30	91	1.21 (0.74-1.98)	0.450
N2	22	111	1.05 (0.61-1.81)	0.862	31	102	1.11 (0.68-1.80)	0.684
N3	54	244	1.16 (0.77-1.76)	0.481	50	248	0.72 (0.48-1.08)	0.112
N (1 + 2 + 3)	97	455	1.12 (0.78-1.62)	0.540	111	441	0.91 (0.65-1.27)	0.573
TNM stage								
I + II	72	389	1.00		103	358	1.00	
III + IV	79	350	1.22 (0.86-1.73)	0.267	81	348	0.79 (0.57-1.10)	0.168
LVI								

No	89	449	1.00		113	425	1.00	
Yes	62	290	1.07 (0.75-1.53)	0.723	71	281	0.92 (0.65-1.29)	0.614
PNI								
No	84	454	1.00		111	427	1.00	
Yes	67	285	1.28 (0.89-1.83)	0.179	73	279	1.00 (0.72-1.41)	0.984
Lauren classification [b]								
Intestinal	44	232	1.00		69	207	1.00	
Diffuse	39	140	1.61 (0.98-2.64)	0.059	31	148	0.70 (0.43-1.14)	0.154
Mixed	39	187	1.11 (0.69-1.79)	0.668	47	179	0.80 (0.52-1.23)	0.309

OR odds ratio, *CI* confidence interval, *LVI* lymphovascular invasion, *PNI* perineural invasion

[a] Adjusted for age, sex, smoking status, drinking status, residence, hypertension, and diabetes in the logistic regression model

[b] The information was not recorded in 209 GC patients.

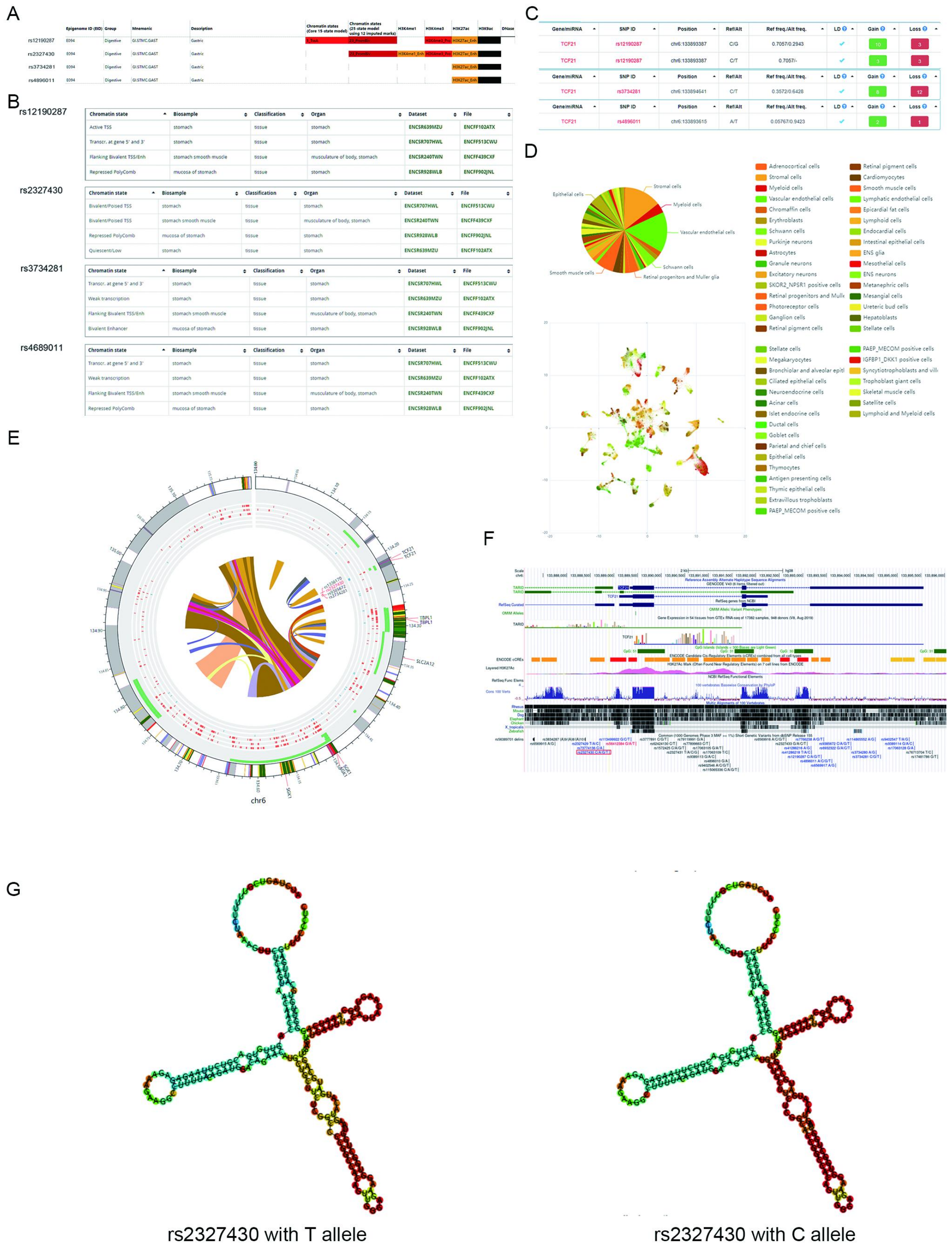

Figure S1 The location of selected SNP sites and the prediction of their function

Notes: (A and B) Regulatory chromatin states from DNAse and histone ChIP-Seq were commented according to Haploreg and RegulomeDB. **(C)** Functions of selected tagSNPs located in 3ʼ UTR regions were predicted by miRNASNP. **(D)** The effect rs2327430 has on the chromatin state was visualized according to the scATAC-seq analysis in the 3DSNP database. **(E)** The circular interaction of rs2327430 was shown in the 3DSNP database. **(F)** The linear plots related to rs2327430 were displayed in the UCSC database. **(G)** The RNA secondary structure of the region near the rs2327430 site was
